# Medicating to practice: a mixed-methods study of Ukrainian psychotherapists in a shared trauma context

**DOI:** 10.3389/fpsyg.2026.1832027

**Published:** 2026-08-06

**Authors:** Oleksandra Martyniuk

**Affiliations:** Department of Psychology, Sigmund Freud University, Berlin, Germany

**Keywords:** psychotherapists, shared trauma, psychotropic medication, armed conflict, clinical ethics

## Abstract

**Introduction:**

Psychotherapists working in armed conflict settings are exposed to a shared reality of war that has been shown to affect their personal wellbeing and professional functioning. Existing literature emphasizes self-care, supervision, and personal therapy as coping strategies, yet these may be insufficient under conditions of prolonged war-related distress. In such contexts, psychopharmacological medication may be used to manage stress and sustain therapeutic practice, but this phenomenon remains largely unexplored. This study investigates the prevalence, motivations, perceived effects, and ethical implications of psychopharmacological use among Ukrainian psychotherapists.

**Methods:**

A mixed-methods design was employed. An online survey (*N* = 103) assessed medication use, war-related exposure, and self-reported effects on mental health and professional functioning. Qualitative data included thematic analyses of five open-ended survey questions and two semi-structured interviews.

**Results:**

More than half of the participants reported current or past use of psychotropic medication, with 41.8% reporting current use and 85.5% continuing clinical work while medicated. Medication use increased markedly after the onset of the full-scale war and participants commonly associated it with war-related stress, grief, and disruptions in emotional and bodily functioning. Participants perceived medication as supporting emotional regulation, therapeutic presence, and boundary maintenance while enabling continued clinical work despite ongoing war conditions. Two interview participants illustrated contrasting patterns of use: (1) reflective, self-monitored medication aimed at sustaining professional functioning and (2) crisis-driven use associated with acute psychological distress.

**Discussion:**

The findings suggest that psychotherapists working in wartime contexts operate within a distinct ethical landscape in which sustaining therapeutic presence and professional responsibility may involve new forms of self-regulation, including psychopharmacological medication. In this context, medication may become part of the efforts to maintain safe and effective clinical practice within a shared trauma environment. These results highlight the need for professional cultures that acknowledge therapist vulnerability and the adaptive strategies required to sustain therapeutic work in humanitarian crises.

## 1 Introduction

Psychotherapists working with trauma are repeatedly exposed to clients' suffering and are, therefore, at risk of secondary traumatic stress, burnout, and emotional exhaustion (Collins and Long, 2003; Figley, 2002). In contexts of armed conflict, however, the psychological burden of therapeutic work exceeds classical models of secondary trauma. When therapists are embedded in the same traumatic reality as their clients, primary and secondary trauma converge into what has been described as *shared trauma* or *double exposure* (Baum, 2010, 2014). Under this phenomenon, therapists are not simply witnesses to trauma but are also simultaneously living through the same ongoing threat, loss, and instability as those they treat. Shared trauma disrupts the foundations of therapeutic work, challenging the expectations of typical clinical settings. In acute shared trauma, the sense of security, understanding, and empathy—normally central to the therapeutic space—becomes entangled with the horror of the events themselves (Tosone, 2008). The boundary between therapist and client often becomes less distinct as personal concerns related to the disaster intrude into therapeutic sessions. Clinicians report heightened emotional engagement, with their usual reflective inner dialog requiring greater effort, sometimes impairing professional functioning. Here, the typical asymmetry and distance in therapeutic relationships diminish, making professional detachment increasingly difficult (Baum, 2010). Personal experiences begin to influence professional performance in profound ways: externally, work responsibilities spill into personal life and personal struggles infiltrate the therapeutic space, while, internally, the separation between professional and personal identity weakens, creating a “gray area” where the two selves overlap (Baum, 2010, p. 255). For some clinicians, the emotional intensity of shared trauma leads them to activate defense mechanisms to manage their heightened sense of vulnerability. This includes desensitization, reduced empathy, limited capacity to contain clients' anxieties, and anger toward patients' expressions of distress (Baum, 2010; Saakvitne, 2002; Tosone et al., 2003). Beyond the therapeutic encounter, clinicians also experience trauma personally as survivors, being affected intrapsychically, interpersonally, and communally and often experiencing lasting changes in self-perception and worldview (Tosone et al., 2012). They may feel personally threatened; worry about the safety of their relatives or close friends; and, as clinicians, be impacted by the distressing material their clients bring into therapy (Saakvitne, 2002; Tosone, 2008). The literature drawn on here and throughout this article has mapped its challenges more thoroughly than it has tested them.

The ongoing war in Ukraine represents a paradigmatic example of a context in which shared trauma may emerge. Since 2014, and with particular intensity following Russia's full-scale invasion in February 2022, the country has been subjected to a sustained military assault that has profoundly disrupted civilian life, infrastructure, and social cohesion. Thousands of missiles and drones have targeted civilian areas, resulting in tens of thousands of verified civilian casualties and widespread destruction of housing, energy systems, and essential services (Kyiv Independent, 2023; Statista Research Department, 2024). The human toll has been intensified by deep personal entanglement with the war: nearly half of Ukrainians report having close relatives serving in the armed forces (Volonter.Org, 2022), while approximately 17% of Ukrainians have lost contact with friends and relatives, leaving them uncertain about their whereabouts and fate (Sociological Group Rating, 2022), and 48.3% reported knowing someone who was killed (Chudzicka-Czupała et al., 2023). In Kyiv alone, by late 2023, 59.5% of residents had experienced combat, 54.5% had endured separation from family, 53.3% had lacked shelter, and 15.6% had grieved the violent loss of a family member or friend (Lotzin et al., 2023). Mass displacement has reached unprecedented levels, with millions internally displaced or forced into refuge abroad (The UN Refugee Agency, 2024; Statista Research Department, 2024). At the same time, the war has precipitated a severe economic collapse marked by widespread job loss, income insecurity, and rising poverty (Chudzicka-Czupała et al., 2023; The World Bank Group, 2023).

Against this background, Ukrainian psychotherapists continue their clinical work while being exposed to air raids, displacement, economic insecurity, and concerns for their own and their families' safety (Goto et al., 2022; Pinchuk et al., 2022). Consequently, wartime atrocities have had a profound impact on the psychological wellbeing of Ukraine's mental health workers: 40% and 44% screen positive for depression and anxiety; 68% report burnout (Pinchuk et al., 2022); and, alarmingly, 10.7% report current suicidal ideation (Kang et al., 2024). This places extraordinary demands on therapists' emotional regulation, professional boundaries, and capacity to sustain a therapeutic presence. Although the literature on trauma work traditionally emphasizes self-care, supervision, and personal therapy as protective factors (Norcross and VandenBos, 2018), these recommendations are largely developed for use within stable, well-resourced contexts. They implicitly assume that therapists can reduce caseloads, take breaks, or temporarily withdraw from practice when distress becomes overwhelming—assumptions that often do not hold true in war-affected settings.

Under conditions of chronic collective trauma, commonly recommended self-care strategies may become insufficient or practically inaccessible. In such circumstances, psychotherapists may turn to additional coping strategies to maintain basic functioning and continue providing care. One such strategy is the use of psychopharmacological medication. This is not a new phenomenon: evidence suggests that health professionals often engage in self-medication due to expectations of resilience embedded in the profession (Montgomery et al., 2011; Myers, 2014). Moreover, mental health professionals' extensive knowledge of psychopathology and treatments renders self-diagnosis relatively accessible (Myers, 2014). It is also known that such behavior is more prevalent in contexts where access to formal mental health care is limited and where over-the-counter availability of medication is common (Rathod et al., 2023). In Ukraine, these structural and cultural factors intersect with wartime conditions, creating a context in which psychopharmacological coping may become normalized rather than exceptional.

The practice of guided medication among psychotherapists has been neither a vocalized nor a well-researched one. International ethics codes in psychology and psychotherapy do not directly equate medication use with impairment. Instead, they require therapists to monitor their fitness to practice and to refrain from clinical work when a mental or other issue may risk causing harm (American Psychological Association, 2017; Canadian Psychological Association, 2017). Moreover, a body of research suggests that therapists' personal experiences of mental issues may serve as a clinical resource rather than solely as a liability. Such so-called “wounded healers” have been shown to provide enrichment to the profession, including deeper empathic connections with their clients, more nuanced clinical judgment, higher levels of patience and tolerance when progress is slow, and greater faith in the therapeutic process (Robertson et al., 2025; Taylor, 2025; Zerubavel and Wright, 2012). The countertransference arising in a wounded healer is also being increasingly recognized as a potential clinical resource, with successful countertransference management being strongly associated with better therapy outcomes. The decisive factor appears to be not the presence or absence of personal emotional reactions but rather the therapist's capacity for self-awareness, self-monitoring, and reflective processing—precisely the capacities that shared trauma conditions demand and strain (Hayes et al., 2018).

However, the line between a distressed wounded healer and an impaired professional is difficult to determine—raising a nearly impossible-to-answer question: who has recovered sufficiently? This tension creates a danger of conflating a medicated therapist with an incapacitated one—promoting the widely existent culture of stigma, conspiracies of silence, and shame around psychotropic medication among psychotherapists. Further, even the studies that advocate for wounded healers metaphorize a wound that can be worked with as a *scar*—something stable enough, old enough, and robust enough to be worked with. An active wound that may “reopen” at any given moment is often viewed in the profession as a slippery slope (Zerubavel and Wright, 2012). However, in war-affected settings, where the psychological toll on clinicians is extreme, shared trauma implicitly present and mental wounding ongoing, this conflation may be particularly harmful, imposing impossible standards, silencing professional conversations, and discouraging help-seeking among those who need support the most.

Consequently, little is known about psychopharmacological (self-)medication among psychotherapists working in active war contexts. The lack of empirical data leaves ethical debates largely speculative and grounded in assumptions derived from Western, non-crisis settings. There is, therefore, a need for context-sensitive research that documents how therapists navigate their own mental health while maintaining professional functioning under extreme conditions. The present mixed-methods study examines psychopharmacological (self-)medication among Ukrainian psychotherapists working during the ongoing war. In an exploratory spirit, and seeking to breach the professional and academic silence around the topic, this study investigates how psychopharmacological medication is used, experienced, and interpreted as a coping and boundary-regulation strategy within a shared trauma context and how it relates to professional functioning in wartime practice. By doing so, the study contributes empirically grounded insights to ongoing discussions on therapist self-care, professional boundaries, and ethical functioning in crisis and humanitarian settings.

## 2 Materials and methods

### 2.1 Study design

This study employed an exploratory approach, documenting self-reported patterns of psychopharmacological (self-)medication among Ukrainian psychotherapists working during wartime. A mixed-methods design integrating quantitative and qualitative data was chosen to capture the prevalence and patterns of medication use and the subjective meaning-making processes through which therapists interpret its role in their professional functioning. Quantitative and qualitative components were assigned equal priority and integrated during data collection and interpretation (Creswell and Plano Clark, 2011).

The quantitative strand consisted of an online survey assessing medication use, contextual factors, and self-reported effects on mental health and professional functioning, with recognition that the cross-sectional, self-report design limits the extent to which causal or normative conclusions can be drawn. The qualitative strand comprised open-ended survey responses and in-depth semi-structured interviews, allowing for an in-depth exploration of motivations, ethical reflections, and boundary management related to medication use in the context of the cultural complexity of Ukrainian reality (Williams et al., 2019).

### 2.2 Data-collection strategy

#### 2.2.1 Survey

The study survey consisted of 49 items, including 44 closed-ended questions and five open-ended questions. Quantitative items assessed demographic and professional characteristics, war-related exposure, psychopharmacological medication use (type, duration, and source of access), continuation of clinical work while medicated, engagement in self-care practices, and access to professional support, along with self-reported effects of medication on stress, emotional resilience, mental health, and professional functioning. Open-ended questions invited participants to elaborate on types of changes in their practice due to war, personal motivations for medication use, and perceived effects of the medication.

#### 2.2.2 Interviews

The semi-structured interview guide comprised 11 open-ended questions addressing participants' professional trajectories, experiences of war-related stress, coping strategies, motivations for medication use, perceived effects on therapeutic presence, ethical concerns, and boundary management. The guide was refined following an initial analysis of survey responses to ensure alignment with emerging quantitative patterns.

### 2.3 Participants and recruitment

#### 2.3.1 Quantitative sample

Participants were eligible if they (a) held Ukrainian nationality and (b) were actively practicing psychotherapy at the time of data collection. Recruitment followed a convenience and snowball sampling strategy. Invitations to participate were disseminated through professional networks, psychotherapy-related social media groups, direct email contact with Ukrainian psychological institutions, and peer referrals. The final sample included 103 participants, whose demographic and professional characteristics are described in Table 1.

**Table 1:** Demographic and professional characteristics of the sample (*N* = 103).

Characteristic	Number of participants	Percentage
73>Location		
Residing in Ukraine	73	70.9%
Living abroad (EU, UK, and US)	30	29.1%
Gender		
Women	91	88.3%
Men	11	10.7%
Non-binary	1	1.0%
Age group		
20–29 years	11	10.7%
30–39 years	35	34.0%
40–49 years	49	47.6%
50–59 years	7	6.8%
≥60 years	1	1.0%
Professional experience		
< 5 years	31	30.1%
5–10 years	45	43.7%
11–20 years	24	23.3%
>20 years	3	2.9%
Work setting and professional roles		
Private practice	91	88.3%
Community-based/NGO	25	24.3%
Clinic-based	8	7.8%
Students in training	3	2.9%
Supervisors	24	23.3%
Trainers	14	13.6%

NGO, non-governmental organization.

#### 2.3.2 Qualitative sample

Following preliminary analysis of the survey data, two interview participants who were affected by war-related events, initiated medication use after the onset of the war, and continued their therapeutic work while medicated (see Table 2) were selected using purposive sampling. This small interview sample size (*n* = 2) was necessitated by the resource constraints of the project but is consistent with the concept of information power, which supports smaller samples when the research aim is narrow, the participants are highly experienced, and the data are rich and relevant (Malterud et al., 2016).

**Table 2:** Demographic and contextual characteristics of the qualitative sample (*n* = 2).

Characteristic	Participant A.	Participant K.
Gender	Female	Female
Age	33 years old	27 years old
Current place of residence	Kyiv, Ukraine	Refugee in Estonia
Academic background	Medical psychologist, specialist diploma	Clinical psychologist, Master's degree, and current PhD student
Therapeutic training	Gestalt therapy (completed); certified supervisor and group therapist	Unfinished trainings in Transactional Analysis and Psychoanalysis
Therapeutic experience	11 years of clinical practice	6 years of clinical practice
Clinical condition	Depression	Generalized anxiety disorder triggered by war trauma
Medication use	Antidepressants; currently finished	Currently on antidepressants

### 2.4 Data analysis

#### 2.4.1 Quantitative analysis

Quantitative data were analyzed using IBM SPSS Statistics (version 30; IBM Corp., Armonk, NY, USA). Descriptive statistics were calculated to examine the prevalence and characteristics of psychopharmacological medication use. Given the exploratory nature of the study and the level of measurement of variables, nonparametric inferential tests were applied. Associations between nominal variables were assessed using the chi-squared test or Fisher's exact test where appropriate. Group differences on ordinal variables were examined using Mann–Whitney *U* tests, and associations between ordinal variables were assessed using Spearman rank-order correlations. Effect sizes are reported alongside inferential tests: *r* (*Z*/√N) for Mann–Whitney *U* tests and Cramér's *V* for chi-squared tests; Spearman's ρ serves as its own effect size. Statistical significance was set at *p* < 0.05 (two-tailed). Non-significant findings are reported only where relevant to the interpretation.

#### 2.4.2 Qualitative analysis

Qualitative data from open-ended survey responses and interview transcripts were analyzed using thematic analysis following Braun and Clarke (2006) six-phase framework, grounded in a constructivist epistemological stance. Analysis operated at a latent level, focusing on underlying meanings and assumptions rather than solely on semantic content. Interview recordings were transcribed using Sonix.ai (Sonix, Inc., Springfield, VA, USA) and manually reviewed for accuracy. Coding and theme development were supported by the MAXQDA software (version 24.9.1; VERBI GmbH, Berlin, Germany). Illustrative quotations were translated from Ukrainian into English for reporting.

### 2.5 Reflexivity

The researcher's positioning as a Ukrainian clinical psychologist trained in Ukraine and currently residing in Germany informed the focus and interpretation of the study. Although this insider position facilitated contextual sensitivity and access to nuanced perspectives, it also necessitated ongoing reflexive awareness of potential biases related to shared cultural and professional experiences of war. Reflexive notes were maintained throughout data collection and analysis to support transparency and critical examination of preconceived ideas (Malterud, 2001; Williams et al., 2020).

### 2.6 Ethical considerations

The study was reviewed and approved by the Ethics Commission of Sigmund Freud University Berlin (approval date: 29 January 2025; project number VDD4MEEUCC3LDD91370). The Commission confirmed that the study does not constitute a clinical trial under the German Medicinal Products Act or Medical Devices Act. Participation was voluntary and anonymous. Survey participants provided informed consent electronically, and interview participants provided written consent prior to participation. All data were stored securely and anonymized. Given the sensitive nature of the topic, participants were provided with information on Ukrainian psychological support services at the conclusion of the survey and interviews.

## 3 Results

### 3.1 Quantitative results

#### 3.1.1 Prevalence and characteristics of psychopharmacological (self-)medication

**Figure 1:**
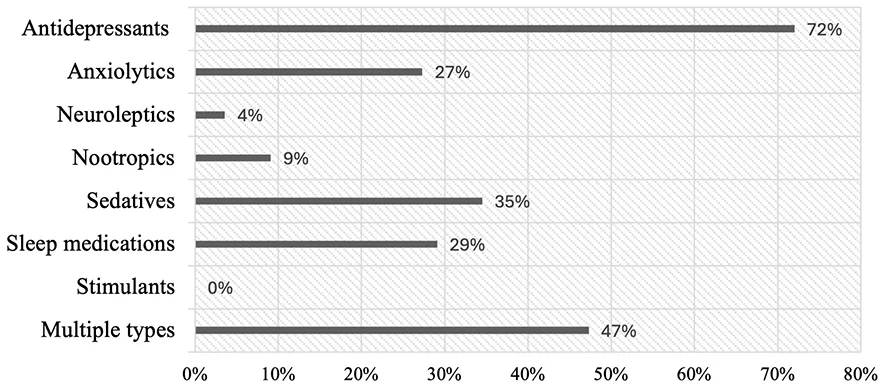
Types of psychotropic medications used by the respondents (*n* = 55).

More than half of the survey respondents (53%, *n* = 55) reported having used psychopharmacological medication at some point in their lives, with 41.8% of these respondents reporting current use of psychotropic medication. When asked about perceived prevalence among colleagues, 66.0% of all participants indicated that psychopharmacological medication use was common among Ukrainian psychotherapists. The most frequently reported medication classes included antidepressants, anxiolytics, sedatives, and sleep medications, with many respondents reporting the use of medications from more than one drug class (see Figure 1).

Among the subgroup that reported past or current use of psychotropic medication (*n* = 55), three participants (5.5%) had used such medication only prior to the 2022 full-scale invasion, 29 participants (52.7%) reported initiating medication use after this escalation, and 23 participants (41.8%) indicated having used psychotropic medication before and after the full-scale invasion. Participants who reported emotional decline related to the war were significantly more likely to use psychotropic medication (*U* = 851.0, *Z* = −3.23, *p* = 0.001, and *r* = 0.32). Cumulative war-related experiences correlated positively with how strongly participants feel triggered by their clients' traumatic reactions [ρ_(103)_ = 0.20, 95% CI [0.00, 0.38], and *p* = 0.043].

Regarding the ways the psychotropic medications were obtained, 62% (*n* = 34) received their medication as part of an official diagnosis, with the medication having been primarily prescribed by psychiatrists (56%), followed by general practitioners (4%) and neurologists (2%). In contrast, 38% (*n* = 21) reported self-sourcing their medications, including buying it without a prescription (16%), asking a colleague to prescribe it (15%), and self-prescribing directly (7%). Among participants who reported current or past medication use, the majority (85.5%) continued to provide psychotherapy while medicated.

#### 3.1.2 Self-reported effects of medication

**Figure 2:**
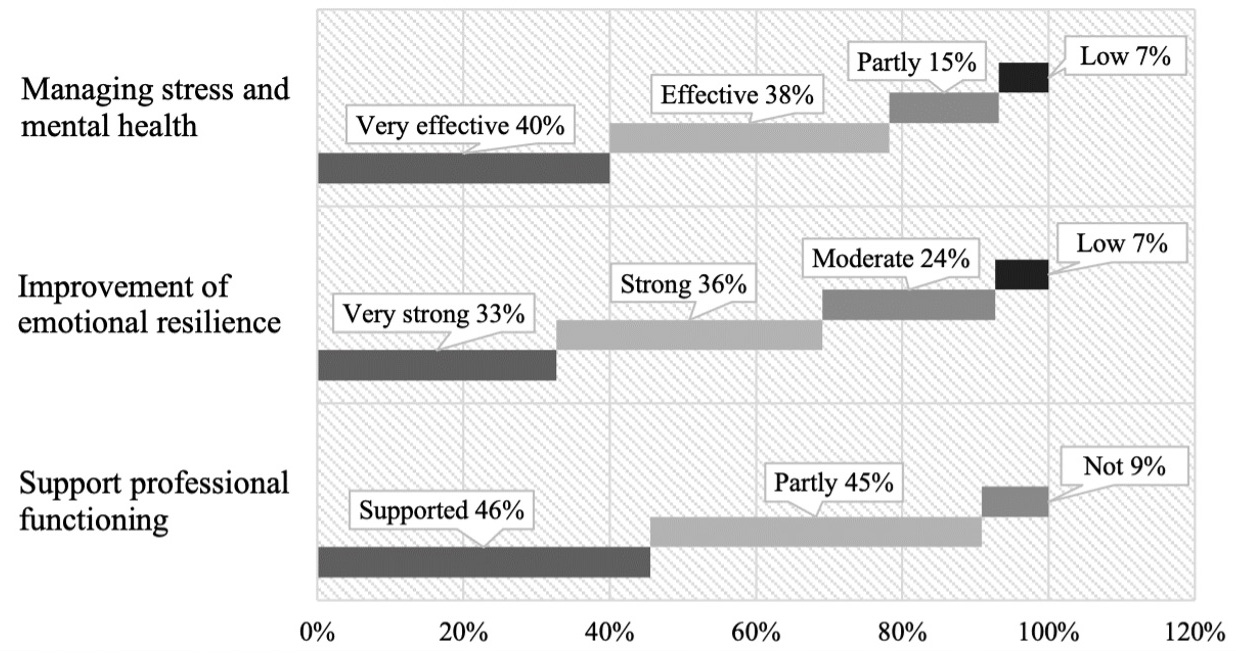
Perceived effectiveness of psychotropic medication for stress and mental health, emotional resilience, and professional functioning (*n* = 55).

Among participants who reported using psychotropic medication (*n* = 55), psychotropic medication was broadly perceived as beneficial. Participants rated medication as moderately to highly effective for managing stress (*M* = 4.11, *SD* = 0.92, and 95% CI [3.86, 4.36] on a five-point scale) and for improving resilience (*M* = 3.95, *SD* = 0.93, and 95% CI [3.69, 4.20]), with no participants selecting the lowest option “not at all.” Nearly all users (91%) reported positive effects on therapeutic functioning (see Figure 2).

In terms of satisfaction with stress management or emotional resilience, no significant differences were found between self-sourced and prescribed medication users. However, participants who obtained psychotropic medication through an official prescription reported higher perceived support for their ability to function as therapists than those who self-sourced their medication, at the conventional significance threshold (*U* = 250.00, *Z* = −1.94, *p* = 0.050, and *r* = 0.26). Participants who obtained psychotropic medication through an official prescription were more likely to feel that the medication supported their ability to function as therapists and more likely to discuss their medication use with supervisors or colleagues (*U* = 247.00, *Z* = −1.99, *p* = 0.047, *r* = 0.27).

### 3.2 Qualitative results

The themes extracted in the original thematic analysis of open-ended survey responses (Transcripts 1–3) and semi-structured interviews (Participant A. and Participant K.) were further pared down to four overarching themes for this study (see Table 3).

**Table 3:** Overview of qualitative themes

Theme	Sub-theme	Sources	Reported *n* times
Theme 1: The war has gravely transformed therapeutic practice	Therapeutic practice has shifted in content (e.g., rise in crisis-oriented work, shift in therapeutic themes, shift in client groups)	Survey, Interviews	149
	Emotional load for therapists has increased		
	The workload has been altered		
	Therapeutic settings have transformed		
	The therapists themselves have experienced transformation (e.g., exhaustion and burnout, posttraumatic growth, and shifts in the moral domain)		
Theme 2: Psychopharmacological medication use as a response to war-induced distress	Stress-induced clinical conditions (e.g., depressive disorders, anxiety disorders, mixed anxiety–depressive states, and adjustment disorders)	Survey, Interviews	68
	Acute grieving		
	Disruptions in bodily and emotional functioning (e.g., apathy, hopelessness, agitation, panic attacks, migraines, bodily tremor, overall weakness, and sleep disturbances)		
	Self-support in critical moments		
Theme 3: Psychotropic medication has helped to restore professional functioning	Return of sleep and improved energy levels	Survey, Interviews	64
	Increased emotional stability		
	Improved therapeutic presence		
	Restoration of therapeutic boundaries		
	Lived experience with medication has become a therapeutic resource		
Theme 4: Taking antidepressants is not a problem—not knowing you need them is		Interviews	2

#### 3.2.1 Theme 1: the war has significantly transformed therapeutic practice

This theme was extracted from the responses of 74 survey participants and both interviewees. Sub-themes are as follows.

##### 3.2.1.1 Sub-theme 1.1: therapeutic practice has shifted in content

Participants reported a marked shift toward crisis-oriented therapeutic work, with an increased focus on grief, depression, trauma, displacement, and acute stress. Client groups have increasingly included veterans, families of combatants, refugees, and internally displaced persons. Participants also noted that the pace of therapeutic change has become noticeably slower, if present at all, concentrating instead on immediate coping.

##### 3.2.1.2 Sub-theme 1.2: therapists' emotional loads have increased

Participants described a pervasive atmosphere of anxiety—their own and that of their clients—along with emotional tension and the need to contain more complex effects, an omnipresent feeling of impotence in the face of the clients' war-related suffering, feelings of shame and guilt when working with particularly disadvantaged clients resulting from the fact that the therapist has better living conditions, and detachment strategies. One survey participant shared the experience of losing her client on the frontline and the immense difficulties of grieving for her. Complementing this account, interview Participant A. provided a visceral, somatic metaphor to describe a painful internal sensation tied to emotional overload: “Inside, it feels like being cut open—and all you want to do is freeze” (lines 113–114).

##### 3.2.1.3 Sub-theme 1.3: the workload has been altered

Workload changes varied substantially across participants. Some began their practice during the war (*n* = 4) or increased their caseloads (*n* = 6), resulting in longer working hours. In contrast, a larger proportion of survey participants (*n* = 11) and both interviewees reported working substantially less, particularly at the beginning of the full-scale invasion. Workload reductions were attributed to clients' financial difficulties and psychotherapists' deliberate efforts to manage emotional strain (“taking [her] foot off the gas,” Participant A., line 96). A small number of survey participants (*n* = 3) reported partially or fully stepping away from clinical work.

##### 3.2.1.4 Sub-theme 1.4: therapeutic settings have transformed

As other changes, participants reported that therapeutic settings have been adapted to shelter-based work or online formats due to the displacement of clients or therapists and payment structures have been altered. Participants also emphasized the need to re-establish trust before engaging in each session of therapeutic work since, in a war setting, “shifts from a friend to foe can sometimes happen very suddenly” (Participant A., lines 627–667).

##### 3.2.1.5 Sub-theme 1.5: therapists themselves have experienced transformations

Participants described pervasive exhaustion, emotional depletion resulting in “numbed empathy” (Transcript 1, line 128), increased fatigue, heightened vulnerability, and a need for extended recovery—especially after missile attacks. As one participant put it, they are “balancing on the edge of burnout” (Transcript 1, line 139). Some survey participants (*n* = 3) described full burnout that forced them to step away from work for more than a year; cognitive decline; and withdrawal from writing, knowledge-sharing, and professional engagement. Similarly, Participant A. reported adopting dissociation and cynicism in her therapeutic work as protective strategies against ongoing fatigue and increased vulnerability.

Conversely, some survey participants reported positive changes described as “posttraumatic growth” (Transcript 1, line 141). This included two main developments: (1) accelerated professional growth, such as acquiring new competences, learning international protocols, deepening theoretical understanding, and gaining skills in trauma and crisis therapy, and (2) strengthened professional identity, with increased confidence and a deeper sense of connection with clients. These positive changes, however, were not reflected in the interviews.

Participants also described shifts in their moral and ethical perspectives. Survey respondents noted their reassessment of personal and professional values, heightened attention to responsibility and justice, and embracing of human suffering as an inherent part of life—alongside a growing awareness of their own limitations. In addition, both interview participants reflected on a painful sense of disappointment in people and the world: “I started believing that the world is […] cruel—like indifferently cruel […] and kind of… broken” (Participant K., lines 190–199).

Finally, Participant K. observed a shift in professional ethics and norms among therapists concerning their own mental health. She noted that the pre-war expectation that a practicing therapist must be “mentally healthy and all that and shouldn't have any diagnoses” (line 664) has largely faded since the onset of the war and been replaced by the acceptance of therapists' reactive mental conditions.

#### 3.2.2 Theme 2: psychopharmacological medication use as a response to war-induced distress

This theme was identified in responses from 51 survey participants and both interviewees. Sub-themes, shown below, reflect how participants conceptualized their difficulties rather than diagnostic precision.

##### 3.2.2.1 Sub-theme 2.1: stress-related clinical conditions

Survey participants named a range of clinical conditions, including anxiety disorders, depressive disorder, mixed anxiety–depressive states, and adjustment disorders. It remains unclear in many cases whether these conditions were formally diagnosed by psychiatrists or are based on participants' self-assessments. The onset of these conditions was frequently associated with specific events, such as the outbreak of the full-scale war, forced displacement, a child's illness, or surviving missile attacks. In these cases, psychotropic medications were used to manage symptoms of the named disorders. Similarly, both interview participants attributed their medication use to a diagnosed condition, reporting anxious–depressive symptoms with suicidal ideation. Participant K. identified the onset of her clinical condition as an acute stress reaction, triggered by the belief that a missile strike had destroyed her childhood home and killed her mother:

“My friend sends me a video of a burning building and asks: ‘K., is that our building?' […] I realize I don't know whether it's ours or not […] My hands are shaking, I'm already panicking. I tried to call my mom; she didn't answer. I called my dad […] and he says, ‘[…] That's not our building.' And I'm thinking, how does he know it's not our building? He doesn't know for sure. He says, ‘Mom's home. It's not our building.' And I think he's just trying to calm me down […] Then my mom calls me, and I pick up, and her voice, it sounded like… like a man's. And the first thing my brain thought was that she'd either been wounded or killed, and someone—like a rescuer—was calling me from her phone… Then it turned out it was my mom” (Participant K., lines 145–164).

##### 3.2.2.2 Sub-theme 2.2: acute grieving

Some participants reported medication use in response to “acute grieving” (Transcript 2, line 78) or “mourning a personal loss” (Transcript 2, line 23), often referring to the death of loved ones.

##### 3.2.2.3 Sub-theme 2.3: disruptions in emotional and bodily functioning

In their responses, participants described emotional symptoms such as apathy, lack of motivation, hopelessness, emotional lability, agitation, and intense sadness. Bodily symptoms were also cited as reasons for initiating medication, including panic attacks, migraines, muscle pain, bodily tremor, overall weakness, and—most prominently—severe sleep disturbances. Sleep disruption emerged as a particularly strong motivator for taking medication, with many participants (*n* = 14) describing persistent insomnia, a complete absence of sleep, frequent awakenings, and early morning waking. The relief associated with psychotropic medication was often linked to the restoration of sleep. As some note: “Physically, I've recovered; mentally, it's more difficult because stress is a constant factor in my life” (Transcript 2, lines 43–44).

##### 3.2.2.4 Sub-theme 2.4: self-management in critical moments

Some participants described situational medication use during periods of acute stress. One participant shared: “I used them situationally at the beginning of the war, when a loved one was fighting on the front line. They helped me respond a bit more calmly” (Transcript 2, lines 36–37).

#### 3.2.3 Theme 3: psychotropic medication helped to restore professional functioning

This theme was extracted from a total of 42 survey responses and both interviewees, focusing on the perceived effects of medication on professional functioning. Sub-themes are presented below.

##### 3.2.3.1 Sub-theme 3.1: return of sleep and improved energy levels

Respondents noted that psychotropic medication helped restore their sleep, representing a fundamental resource with a direct influence on their overall functioning and professional capability. As one participant expressed: “I just started getting enough sleep, and it affected everything—my wellbeing, energy levels, and ability to work” (Transcript 3, lines 53–54). In some cases, the restoration was minimal: “I was able to get out of bed every morning and maintain personal hygiene” (Transcript 3, line 9) or “I was simply able to work” (Transcript 3, line 45). Others described more substantial gains, such as feeling less tired, returning to full workloads, accepting new clients, or enrolling into new studies.

##### 3.2.3.2 Sub-theme 3.2: increased emotional stability

Medications were reported to reduce anxiety, emotional lability, irritability, helplessness, apathy, and guilt. Participants described feeling more motivated, patient, compassionate, and optimistic in their work. Some noted that medication renewed their interest in clients and restored their capacity for empathy. However, a few participants (*n* = 2) acknowledged a numbing effect, stating that, “at the same time, it reduced sensitivity to emotions” (Transcript 3, line 17). Participant A. reflected on the long-term stabilizing effects of her medication, describing it as having built a “resilience reserve” (line 234) that helped her cope with the initial shock and disruption of war. Participant K. reported a return of optimism and the capacity to experience joy—in relation to her personal accomplishments and in response to her clients' therapeutic progress.

##### 3.2.3.3 Sub-theme 3.3: improved therapeutic presence

One's own emotional stabilization was linked to restored therapeutic presence and resilience—achieved through psychotropic medication. This included a greater capacity to attune to clients' needs, tolerate intense affect, and maintain the emotional stability to be able to explore deep and painful topics. One survey participant described this as the ability to “be resilient and supportive—both for myself and for others” (Transcript 3, line 25). Others emphasized cognitive benefits such as enhanced concentration, attention, and memory that contributed to more effective therapeutic engagement and “helped [them] focus on the contact with the client” (Transcript 3, line 55).

##### 3.2.3.4 Sub-theme 3.4: restoration of therapeutic boundaries

Both survey and interview participants noted that the overarching effect of psychotropic medications resulted in them being “better able to separate my professional practice from my personal life” (Transcript 3, lines 35–36). They reported getting less triggered by their clients' stories, developing the emotional capacity to process clients' experiences rather than being absorbed by their own, and confronting clients about their counter-adaptive worldviews. As one survey participant noted: “I no longer sit with them thinking that this world has no hope for a better future. Instead, I focus on what is in their life that makes them think this way” (Transcript 3, lines 43–44).

##### 3.2.3.5 Sub-theme 3.5: lived experience with medication became a therapeutic resource

Interview participants described their medication history as more than self-care—as a part of their clinical toolbox, enabling them to relate more effectively to their clients' fears and normalize help-seeking. Participant A. noted:

“With a few clients, the alliance really got stronger in that exact spot, because they were also taking antidepressants, or wanted to but were scared. And it kind of became a shared point—like I had this experience I could lean on, something I could speak from. I started to understand that resistance better—because it also took me a long time to get here. I knew what exactly could be scary, how it might feel inside, how people can adapt, what kinds of changes happen” (Participant A., lines 307–313).

#### 3.2.4 Theme 4: taking antidepressants is not a problem—not knowing you need them is

This theme recurred across the dataset and in cross-case comparisons between interview Participants A. and K. Both participants rejected the idea that using medication is incompatible with good therapeutic work. Instead, they saw medication as compatible with ethical and effective clinical work and contributing to it—as long as it's accompanied by self-reflection and awareness.

**Figure 3:**
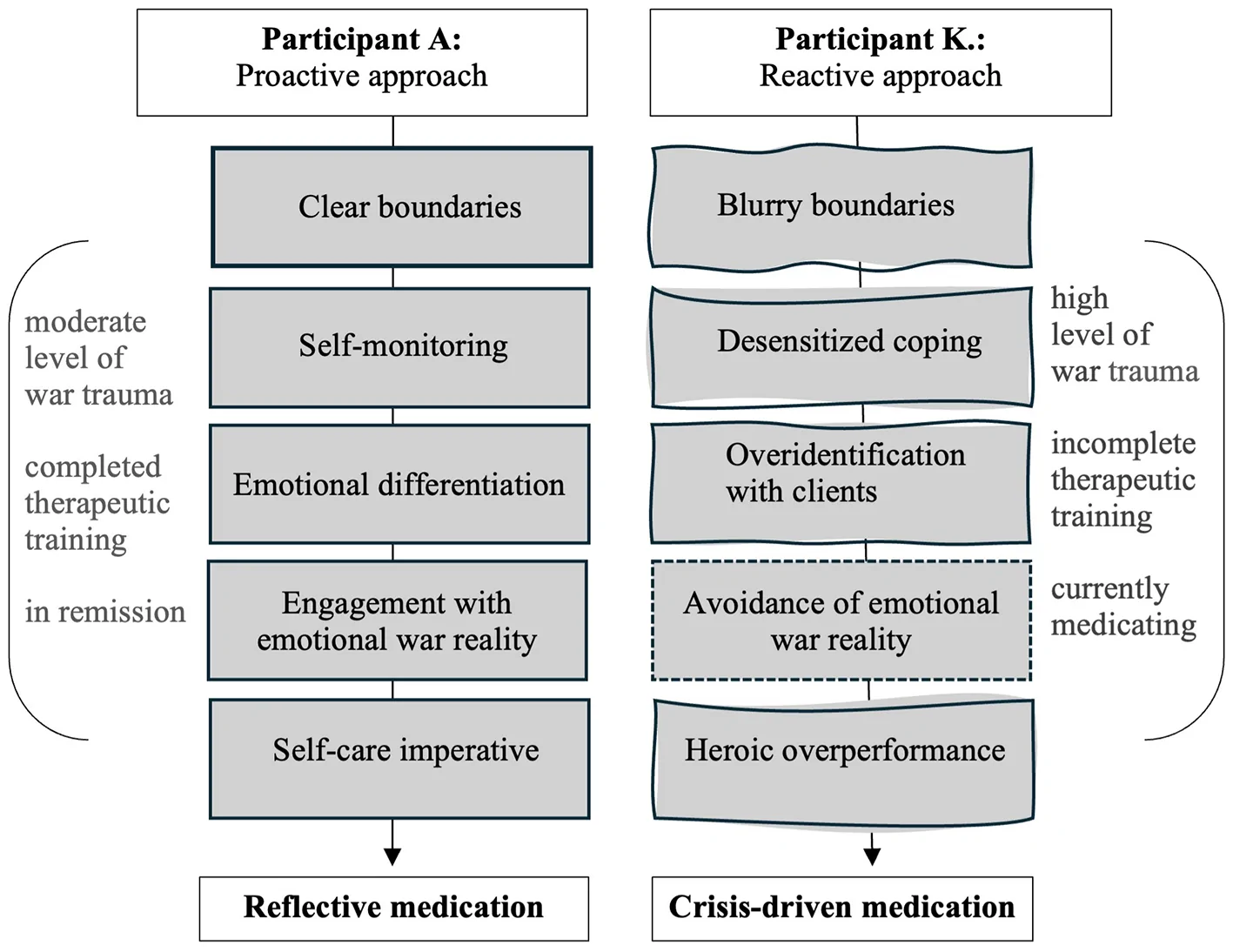
Contrasting illustrations of therapeutic functioning and medication use.

However, the two interviewees displayed diverging strategies of navigating the boundaries of their practice on medication (see Figure 3). These two accounts are presented as individual illustrations, not as a typology of medicated practice. Participant A. described a self-aware approach, where boundaries were consciously monitored and enacted as an ethical imperative. She used emotional and embodied self-monitoring (e.g., eating patterns, loss of meaning, suicidal ideation, and emotional numbness as warning signs) as well as regular supervision to delineate the limits of her therapeutic capacity. When she noticed herself becoming overloaded, she would reduce the workload for the sake of her own and her client's safety. In this frame, her approach to medication was reflective: motivated by care and based on a nuanced, early-warning system. She explained:

“I notice that I'm no longer fit for therapy, like when I can't remember things about my clients, or I can't stay present with them for the full 50 mins [...], when I start noticing that this is becoming systematic—and it's not about the clients' dynamics, but something in me that just can't stay in contact—that's when I know I need to stop” (Participant A., lines 429–442).

“If my eating habits become completely dysregulated [...] or this sense of meaninglessness doesn't go away, and I start getting more intrusive suicidal thoughts. [...] But it's like… I don't quite get to that point—I get close. I kind of stay in the yellow zone, maybe put one foot into the orange, but I don't cross into it” (Participant A., lines 286–294).

In contrast, Participant K. described a more reactive course, marked by blurred boundaries, delayed self-recognition, emotional overwhelm, and overidentification with clients. She displayed “heroic overperformance” as a caregiver that led to an unrecognized overload, defensive emotional numbing valorized as a coping achievement, and eventual internal collapse. In this context, her decision to initiate medication emerged not as a result of self-monitoring and reflective boundary-setting but as a belated response to a total functional collapse. Even then, however, she did not pause her practice:

“Through my work, I'm trying to contribute to the wellbeing of humanity. And I give, like, literally everything I've got” (Participant K., lines 184–186).

“I really should have taken, like, a week off—a pause. But I didn't realize it at the time [...], but all my clients noticed that something was wrong with me. All of them. [...] I feel sad for myself, that I had to let it get to a point where I was 100% not okay. [...] My brain had officially checked out, totally gone… And now let's go—give me the pills, give me the whatever, I am definitely not okay” (Participant K., lines 497–504 and 588–593).

## 4 Discussion

### 4.1 What is happening: normalization of guided medication use

More than half of the surveyed psychotherapists reported current or past use of psychotropic medication as a self-regulatory strategy to manage stress or maintain mental health, with 41.8% of them taking medication at the time of the study. Two-thirds believed that psychotropic use is common among their peers, and antidepressants were the most commonly reported drug class in use. This prevalence is consistent with broader population trends.Globally, psychotropic medication consumption has risen steadily, increasing at an average annual rate of approximately 4% between 2008 and 2019 (Brauer et al., 2021). In Europe specifically, antidepressant use increased over the past decade, ranging from 6% to over 150% increase depending on the country (Bindel and Seifert, 2026). For example, approximately 9% of the Swiss adult population was shown to receive at least one antidepressant prescription, with the proportion of long-term users steadily increasing (Amrein et al., 2023). Against these statistics, psychotropic medication use among therapists is neither exceptional nor unique to the profession—it reflects a broader societal reality in which pharmacological management of mental health conditions has become commonplace. What is exceptional is the persistent expectation that therapists remain exempt from this reality.

However, the results of this study indicate that the stigmatizing assumption of a non-medicated therapist is challenged in the Ukrainian therapeutic community. The fact that 85.5% of those using medication continued their clinical work, together with the theme “*Taking antidepressants is not a problem—not knowing you need them is*,” suggests that medication use is not only widespread but also increasingly normalized within the therapeutic profession during wartime. Medication in this study was not viewed as incompatible with therapeutic functioning or professional ethics: both interview participants explicitly rejected the notion that medication compromises therapeutic competence and even described it as enhancing their clinical presence. In line with this, the predominance of antidepressants, anxiolytics, sleep medications, and sedatives in this study aligned with the symptom profiles reported by participants, including stress-related conditions, acute grief, and disturbances in emotional and bodily functioning, and these medications were further reported by a striking 91% of survey participants as positively affecting therapeutic functioning. This view of professional fitness challenges the stigmatizing assumption that a medicated therapist is automatically an impaired one.

The discussed normalization is further complicated by how therapists access medication. More than a third of medication users obtained psychotropic drugs through informal or self-sourced channels, highlighting a gray zone between formal psychiatric care and self-managed coping. This aligns with previous research documenting high levels of self-medication among medical and mental health professionals (Albawardi et al., 2024; Montgomery et al., 2011; Vergès et al., 2022). Such practices have been linked to professional cultures that discourage clinicians from assuming the patient role and emphasize self-reliance and autonomous treatment, often reinforced by stigma and perceived incongruence between vulnerability and professional identity (Montgomery et al., 2011). In Ukraine, these dynamics are intensified by systemic barriers, including economic hardship, underfunding, and infrastructural disruptions that limit access to formal psychiatric care (Chudzicka-Czupała et al., 2023; Weissbecker et al., 2017). At the same time, psychotropic medications are often readily available over the counter, a pattern common in many low- and middle-income countries (Rathod et al., 2023). When combined with the energy depletion and cognitive slowing widely reported by participants, these conditions reduce the capacity to engage with complex or emotionally demanding systems of care, rendering self-medication a more accessible alternative.

Self-sourcing behaviors, however, prevalent, do not appear to be normalized in this study. The finding that those having medications officially prescribed as part of their treatment and not self-sourced were more likely to discuss their medication use with supervisors or colleagues may indicate that self-prescription remains deviant from professional ethical discourse and is possibly accompanied by greater feelings of shame. Meanwhile, the perception that formally prescribed medication better supports professional functioning than self-prescribed use raises concerns about potential inaccuracies in dosing or management among those self-medicating. Taken together, the findings suggest a clear distinction within Ukrainian therapeutic culture: guided pharmacological treatment appears normalized and professionally integrated, whereas self-prescription continues to constitute an ethical boundary violation, consistent with international professional standards (e.g., Lukovic et al., 2014; Norcross and VandenBos, 2018).

### 4.2 Why it is happening: shared trauma as a context in which the need for medication emerges

The escalation of the Russia–Ukraine war itself emerges as a central pivot point in medication use among Ukrainian therapists. The fact that more than half of all users initiated medication after the onset of the full-scale war whereas only 5.5% had used such medication exclusively prior, combined with the finding that therapists who reported emotional decline due to the war were significantly more likely to initiate medication, suggests that psychopharmacological use is not merely a personal coping strategy but also a response to the psychological disruptions induced by war. This interpretation is supported by qualitative findings: participants frequently linked the onset of their clinical symptoms and their corresponding medicating behavior to specific war-related events such as the initial outbreak of the full-scale war, forced displacement, or surviving missile attacks. Moreover, the normalization of the use of medication to support therapists' mental health and its integration into the professional discourse were observed specifically after the escalation of the war, as reflected in one of the interviews.

This turn to medication must be understood within the broader reality of a collective shared trauma. Therapists in this context are not functioning as neutral professionals—they are experiencing a dual exposure to trauma (Baum, 2010), being simultaneously survivors of war and caregivers and emotional containers for their clients, who are affected by the same war. Within this shared trauma landscape, the overarching qualitative theme “the therapists themselves have experienced transformation” emerged, reflecting changes in emotional functioning, worldview, and professional experience in response to sustained war-related distress that are consistent with trauma frameworks. Although no diagnostic assessment was conducted, reported experiences of low mood, apathy, emotional exhaustion, hypervigilance, avoidance, and insomnia strongly overlap with trauma-related response patterns of posttraumatic stress disorder and complex posttraumatic stress disorder (World Health Organization, 2024a,b). The described symptoms of feeling depleted, emotionally numbed, cynical, or desensitized align with the secondary trauma models of compassion fatigue (Figley, 1995) and burnout (Maslach and Leiter, 2016). Some also reflected on aspects of posttraumatic growth, a known response to adversity that can coexist with suffering (Powell et al., 2003; Arnold et al., 2005). Finally, shifts in the moral domain have been reported, such as a painful sense of disappointment in people and the world, that may indicate the presence of moral injury, as described by Shay (2012) and Litz et al. (2009).

At the same time, therapists are working with clients who have been equally or more affected, and their clinical practice was found to have undergone substantial transformation. Therapeutic content has shifted, with an increase in war-related themes and a rise in crisis-oriented work. Client populations have changed as well, with more refugees, veterans, and others directly impacted by the war. The emotional intensity of sessions has increased, while therapy settings have become more fluid, unstable, or remote. Flowing from the shared trauma reality, it is only logical that, in this study, therapists with greater personal war exposure felt more emotionally triggered by clients' trauma responses. This finding is also consistent with well-established findings showing that a therapist's personal history of trauma significantly increases their vulnerability when working with traumatized clients (Baird and Kracen, 2006; Baruah et al., 2024; Figley, 1995, 2002).

Taken together, this study suggests that war disruptions and the shared trauma context serve collectively as a central force generating intersecting forms of distress and creating psychological conditions that, for many, feel unmanageable without pharmacological support.

### 4.3 How medication functions clinically: medication as a containment strategy

It has been discussed that the use of psychotropic medication among therapists in Ukraine is highly prevalent and appears to be a response to the distressing war reality. In this context, participants described medication as a valuable resource, reporting its effectiveness in managing stress, enhancing emotional resilience, and perceived restoration of therapeutic functioning. Qualitative data elaborated on these effects, including the self-reported return of sleep and energy, improved affect regulation and emotional stability, strengthened therapeutic presence, and the perceived restoration of professional boundaries. These benefits were often framed as compensating for psychological deficits caused by the war, allowing therapists to stabilize and continue working under extreme conditions. Given that shared trauma is known to blur therapeutic boundaries and activate defensive responses such as desensitization and reduced capacity to contain clients' intense affects (Baum, 2010; Saakvitne, 2002; Tosone et al., 2003), the findings suggest that psychopharmacological medication may function as a stabilizing containment resource within this context.

A further benefit emerged in a sub-theme “*Lived experience with medication becomes a therapeutic resource*”: Therapists perceived their own medication use as enabling them to more empathically accompany clients navigating their own psychopharmacological journeys. This finding may be particularly valuable given that many therapists feel ill-equipped or anxious when working with medicated patients and often lack sufficient knowledge of psychotropic medications (Blair et al., 2021). It also resonates with the wounded healer literature, which suggests that reflectively processed lived experience of struggles can enhance rather than compromise therapeutic practice (Taylor, 2025; Zerubavel and Wright, 2012). At the same time, it is important to emphasize, that all of these reported effects derive from therapists' own accounts and, therefore, document how therapists perceive and interpret medication's role in their work, but cannot establish whether medicated practice was in fact clinically more effective.

It would be particularly valuable to compare findings of this study with existing literature on how clinicians perceive their own practice while on psychotropic medication—yet, to date, there appears to be little to no research on the matter. This silence likely reflects enduring professional stigma around clinicians practicing while medicated, concerns about ethical judgment, and possibly an underlying Western assumption that therapists do not practice at all when their mental stability is compromised to the extent that medication is needed.

In contrast to the stigmatizing and unrealistic expectation that therapists must be paragons of mental health, a humanizing, culturally sensitive lens is needed. In accordance with a sociopolitical view on trauma (Becker, 2021; Summerfield, 2001, 2002), psychological distress—and the use of medication to manage it—should not be pathologized or treated as a personal failure but rather recognized as a normal response to extreme, abnormal conditions. Within the Ukrainian context, the pursuit of impeccable psychological stability is not merely unrealistic but also structurally constrained by ongoing societal demands. Moreover, the option of withdrawing from professional responsibilities—for instance, via a sabbatical—is often practically unavailable. The economic instability in Ukraine, which has significantly worsened since the start of the full-scale war (The World Bank Group, 2023), is further supported by survey and interview data (e.g., reductions in client hours due to the war) and creates a push for therapists to keep working and preserve their income. In addition to that, the numbers of psychotherapeutic staff in Ukraine are very limited due to systemic conditions (Weissbecker et al., 2017) and war-related disruptions (Goto et al., 2023; World Health Organization, 2025).

Medication, in this context, becomes a bridge between inner vulnerability and the omnipresent necessity of staying functional. Within this reality, and in light of the perceived positive effects of medication on clinical practice in this study, psychotropic medication emerges as a realistic stabilizing force that may allow therapists to uphold an ethical practice where they feel more emotionally stable, present, and able to maintain boundaries.

### 4.4 What is the boundary: Ukrainian therapists renegotiating ethical practice

Although the findings of this study point to a different reality of practice amid shared trauma and to perceived benefits of medicated practice, it is also important to consider under which conditions such practice may be safe and ethical.

As revealed in the data, Ukrainian therapists have reported many symptoms of distress, possibly exhibiting signs of various types of traumatization. Regarding the working stance, the interview data suggested an omnipresent dissociation and emotional desensitization, as well as overidentification with clients and emotional overwhelm in response to the unrelenting psychological toll of living and working through war. Upon this background, the arising ethical questions are: how can therapists practice ethically and effectively when they are, as the data suggest, highly distressed and actively wounded? Also, what guidelines do Ukrainian therapists follow under these extraordinary conditions?

One possible answer is suggested by the contrasting approaches of the two interviewees. While one participant relied on active emotional tracking, self-care, and reflective medication use, the other coped through avoidance, overidentification, and reactive medication use. In this context, the approach to medication—whether based on reflective monitoring or crisis-driven collapse—appears to be a consequence and indicator of how well-boundaries are maintained. Where self-care and boundaries of practice were seen as an imperative, psychopharmacological support could be used thoughtfully—or not be needed at all. Conversely, where boundaries collapsed under the weight of shared trauma and compulsive caregiving, medication became a patch for systemic emotional depletion—a double-edged sword that restores professional boundaries while revealing how far they have already eroded. Building on this contrasting illustration, the present study suggests that medication amid shared trauma of war may be embedded in an ethical practice, only if one recognizes that self-reflection may be impaired at any moment by war-induced desensitization and dissociation—and in maintaining constant, real-time awareness of one's emotional state.

Ultimately, this study suggests that Ukrainian therapists in wartime are actively humanizing the professional culture: they separate the fact of being medicated from the quality of their work, viewing guided medication as a legitimate tool within an ongoing ethical effort to sustain safe and effective practice. What emerges from these data as the markers of ethical practice amid war—medicated or not—are the therapist's awareness of their limitations, a proactive self-care approach, real-time self-monitoring, responsiveness to external feedback, a willingness to seek psychiatric oversight, and a readiness to pause or adjust their practice if their ability to function ethically becomes compromised.

### 4.5 Clinical and ethical implications

The findings challenge the prevailing professional assumption that acute woundedness, along with the psychotropic medication use resulting from it, is incompatible with competent therapeutic practice—an assumption that, while not codified in ethics codes, continues to shape professional culture and discourage help-seeking among clinicians. Ukrainian psychotherapists are currently working under conditions that profoundly shape their clinical roles, emotional stability, and capacity to provide care, making ethical functioning inseparable from the broader realities they face. Under such conditions, ethical practice cannot be meaningfully evaluated using standards developed for stable, low-risk environments; instead, context-sensitive ethical frameworks are needed that recognize the potentially stabilizing role of psychopharmacological support.

Medication use in high-stress contexts should, therefore, not be equated with professional impairment or disqualification. Rather, as therapists themselves perceive it, it may represent an ethically responsible adaptation supporting therapeutic presence, emotional availability, and continuity of care. The central ethical distinction that emerges is not between medicated and unmedicated therapists but instead between those who are reflective, supported, and responsive to their limits and those who are untreated, isolated, and at risk of collapse, rendering binary notions such as “fit vs. unfit to practice” insufficient.

The findings also call for a shift away from heroic, self-sacrificing models of practice toward recognizing limitations as ethical responsibilities rather than failures. This shift requires adaptations in training and supervision for trauma workers, including explicit attention to vulnerability and the realities of shared trauma contexts. Ethical functioning under such conditions cannot rely solely on individual self-regulation but instead depends on robust collective structures of support. It further entails normalizing therapist vulnerability within mental health infrastructures, including ensuring access to psychiatric consultation and psychopharmacological support for clinicians themselves.

Finally, this study highlights a glaring absence of empirical research on how therapists function while on medication. These findings are among the first to bring clinician perspectives on medicated practice into focus. Further research is urgently needed to document, destigmatize, and theorize the role of psychopharmacology in therapists' professional lives—particularly in high-stress and humanitarian settings.

### 4.6 Limitations and suggestions for future research

Several limitations should be considered when interpreting these findings. First, the study relied entirely on self-reported data without validated instruments. Professional functioning, stress, and resilience were assessed through participants' subjective evaluations rather than through standardized measures. The findings of positive effects of medication on therapeutic functioning, therefore, reflects perceived benefit rather than objectively measured improvement. This design choice was consistent with the exploratory aims of the present study, which prioritized ecological validity and contextual sensitivity in a setting where standardized instruments developed for stable, Western contexts may not fully capture the realities of wartime practice. Nevertheless, future research would benefit substantially from incorporating such measures.

Second, the qualitative component involved only two interview participants. While this is consistent with the concept of information power (Malterud et al., 2016), the contrasting approaches to medication use observed in these interviews should be understood as individual illustrations rather than as established trajectories. Whether the patterns identified here represent broader tendencies among medicated therapists is a question for future, larger-scale qualitative research.

Third, the convenience and snowball sampling strategy likely introduced selection bias, with probable overrepresentation of therapists who are psychologically engaged, willing to reflect on sensitive topics, or more distressed than the broader population of Ukrainian psychotherapists. The true prevalence of psychotropic medication use among Ukrainian therapists as a whole remains unknown.

Fourth, the current study did not include client perspectives, patient outcome data, or external assessments of therapeutic competence. Claims about the effects of medication on professional functioning are, therefore, limited to the therapist's own perspective. Future research that incorporates client-reported outcomes, observer-rated session quality, and longitudinal tracking of therapist wellbeing and clinical effectiveness would substantially strengthen the evidence base.

Finally, comparative studies across different conflict-affected or high-stress settings could help determine the extent to which these findings generalize beyond the Ukrainian context.

## Data Availability

The raw data supporting the conclusions of this article will be made available by the authors, without undue reservation.
